# 186. *Klebsiella aerogenes*: Are there implications to taxonomic accuracy?

**DOI:** 10.1093/ofid/ofac492.264

**Published:** 2022-12-15

**Authors:** Lauren Nicholas Herrera, Matthew L Brown, William S Edwards, Sixto M Leal, Joshua Stripling, Rachael A Lee

**Affiliations:** Vanderbilt University Medical Center, Tennessee; University of Alabama at Birmingham, Birmingham, Alabama; University of Alabama at Birmingham, Birmingham, Alabama; University of Alabama at Birmingham, Birmingham, Alabama; University of Alabama at Birmingham, Birmingham, Alabama; University of Alabama at Birmingham, Birmingham, Alabama

## Abstract

**Background:**

With advances in genomics, microbiologists can accurately rename existing bacteria which may cause confusion among clinicians familiar with prior names. Limited investigation is available on the clinical impact of these name changes. University of Alabama at Birmingham Hospital microbiology updated *Klebsiella aerogenes* in the electronic medical record (EMR) to "*Klebsiella* (*Enterobacter*) *aerogenes*" to reflect change in nomenclature. Therapy selection differs between *Klebsiella* pneumoniae and *Enterobacter* spp. due to differences in Ambler Class C (AmpC) beta-lactamase production. Our Antimicrobial Stewardship Program previously implemented selective and cascaded susceptibility reporting for *E. aerogenes* to guide antibiotic selection which remained after nomenclature change. The goal of this study is to determine if the change led to inappropriate antibiotic use.

**Methods:**

We reviewed blood culture data from May 2016-2020 which grew *Enterobacter aerogenes* or *Klebsiella* "*Enterobacter*" *aerogenes*. We excluded polymicrobial blood cultures, patients aged 18 and under, and patients who died within 48 hours of drawing blood cultures. Appropriate therapy was defined as an antibiotic with reported susceptibility with consideration of AmpC induction for a duration appropriate to clinical syndrome with minimum of 7 days. We recorded patient demographics, nature of therapy, and clinical outcomes. We performed comparative analysis with descriptive statistics to compare the two groups.

**Results:**

There were 38 patients with *K. aerogenes* bacteremia, 21 patients prior to name change and 17 afterwards. None were ESBL. Cefepime was most often used directed inpatient therapy while oral ciprofloxacin was the most common outpatient regimen with no use of ceftriaxone. Prior to name change, there was one case (4.76%) that did not receive targeted AmpC therapy while there were two afterwards (11.8%), one of which decided by the ID consult team.

**Table 1:**
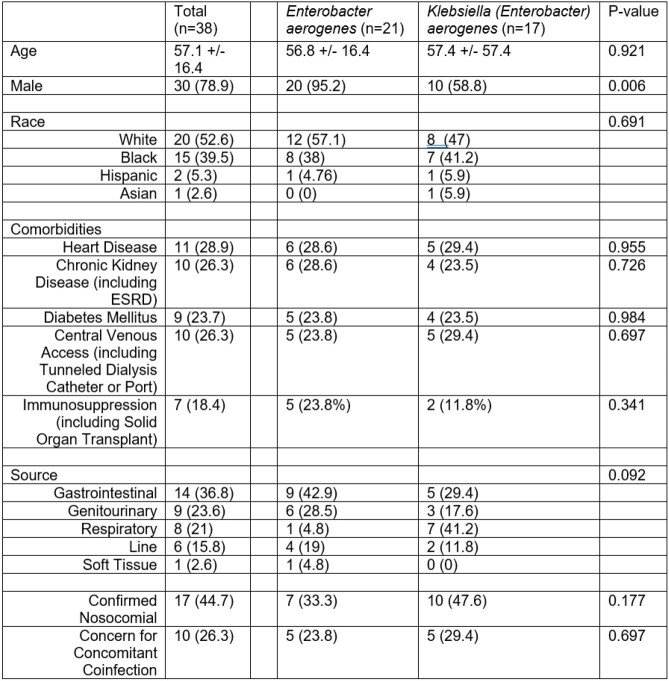

Demographics (Data presented as No. (%))

**Table 2:**
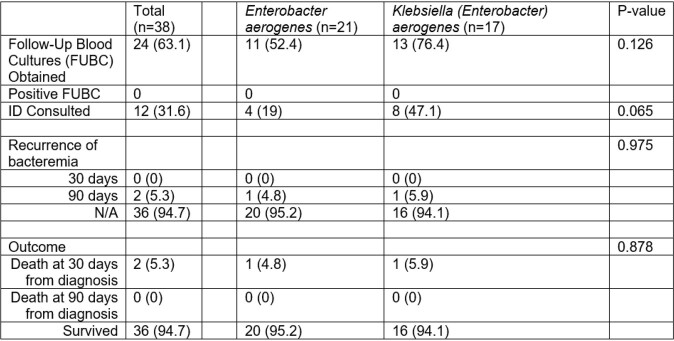

Microbiology and Outcomes (Data presented as No. (%))

**Table 3:**
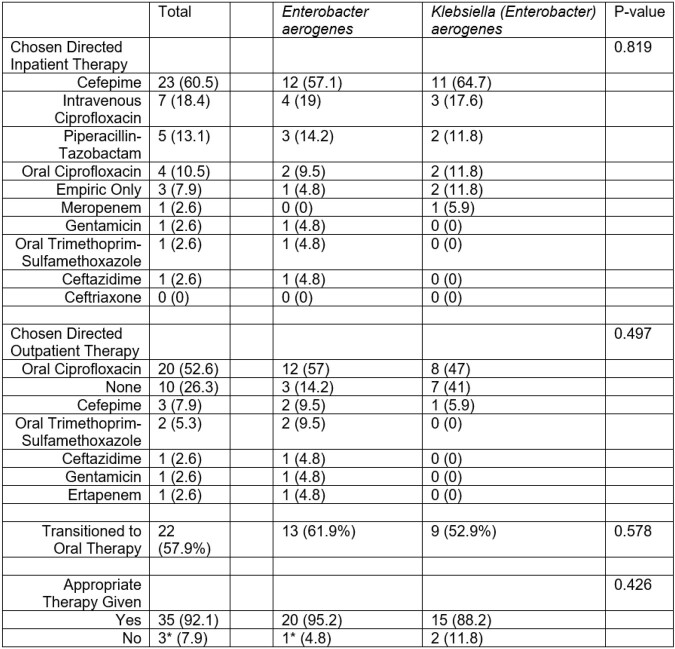

Antimicrobial Selection. One patient that received 72 hours of appropriate therapy but died prior to completion of therapy. This was labeled labelled as appropriate therapy.

**Conclusion:**

Care must be taken when taxonomic changes of clinically relevant bacteria may potentially affect optimization of antibiotics. As the ID consultants, we must be aware of these name changes and provide up to date recommendations. Cascade reporting and documentation in the EMR may be helpful to reduce morbidity related to changes in nomenclature.

**Disclosures:**

**Sixto M. Leal, Jr., MD, PhD**, GenMark Dx: Grant/Research Support|GenMark Dx: Honoraria.

